# Menke–Hennekam Syndrome as a Domain-Resolved Disorder of the Transcriptional Coactivators CBP and p300

**DOI:** 10.3390/genes17101285

**Published:** 2026-10-15

**Authors:** Despina Sanoudou, Anna Maria Anastasiou, Aristides G. Eliopoulos

**Affiliations:** 1 Clinical Genomics and Pharmacogenomics Unit, and Genetic and Pharmacogenetic Counseling Clinic, 4th Department of Internal Medicine, School of Medicine, National and Kapodistrian University of Athens, 11527 Athens, Greece; amanastasiou@hotmail.com; 2 Biomedical Research Foundation of the Academy of Athens, 11527 Athens, Greece; 3 Molecular Genetics Unit, Department of Biology, School of Medicine, National and Kapodistrian University of Athens, 11527 Athens, Greece; 4 Institute of Molecular Biology and Biotechnology, Foundation for Research & Technology-Hellas, 71003 Heraklion, Greece

**Keywords:** Menke–Hennekam syndrome, *CREBBP*, *EP300*, CBP/p300, chromatinopathy, DNA methylation, episignature, neurodevelopment, transcriptional rewiring, iPSC, organoids, genetics

## Abstract

Menke–Hennekam syndrome (MKHK) is a rare neurodevelopmental disorder caused by pathogenic variants in discrete regions of the transcriptional coactivators CBP and p300, encoded by *CREBBP* and *EP300*. Recent genotype–phenotype and DNA methylation studies support a domain-resolved classification involving their ZZ, TAZ2 and ID4 regions and distinguish MKHK from classical Rubinstein–Taybi syndrome. Here, we review the genetic and clinical architecture of MKHK, the regulatory functions of the affected CBP/p300 domains and the emerging epigenomic taxonomy of the disorder. We discuss emerging evidence that localized variants may alter specific protein interactions, chromatin engagement or inter-domain regulation rather than uniformly reducing coactivator activity. On this basis, we propose domain-selective transcriptional rewiring as a testable framework for MKHK pathogenesis. We further consider experimental models required to establish causal mechanisms and implications for therapeutic development. MKHK provides an informative example of how pathogenic variants in multidomain regulatory proteins can produce domain-specific developmental disorders.

## 1. Introduction

*CREBBP* and *EP300* encode CBP and p300, respectively, that are closely related members of the lysine acetyltransferase 3 (KAT3) family of transcriptional coactivators with important roles in gene regulation, chromatin function and development [1]. Pathogenic variants in these genes are classically associated with Rubinstein–Taybi syndrome (RSTS), most commonly through loss-of-function mechanisms leading to haploinsufficiency [2,3,4,5,6]. However, a decade ago, Menke et al. described 11 individuals carrying de novo missense variants within the distal region of exon 30 or proximal exon 31 of *CREBBP* with a neurodevelopmental phenotype clinically distinct from RSTS [7] (see also Supplementary Table S1). In particular, these individuals lacked broad thumbs and halluces and the characteristic RSTS craniofacial gestalt. Recognition of a comparable phenotype associated with homologous variants in *EP300* subsequently established Menke–Hennekam syndrome (MKHK; OMIM #618332 and #618333) as a disorder involving both CBP and p300 [8,9,10,11,12]. The initial distinction between MKHK1 and MKHK2, based on the involvement of *CREBBP* or *EP300*, respectively, provided a useful genetic classification but did not account for the marked clinical heterogeneity that was subsequently recognized within the disorder.

**Figure 1:**
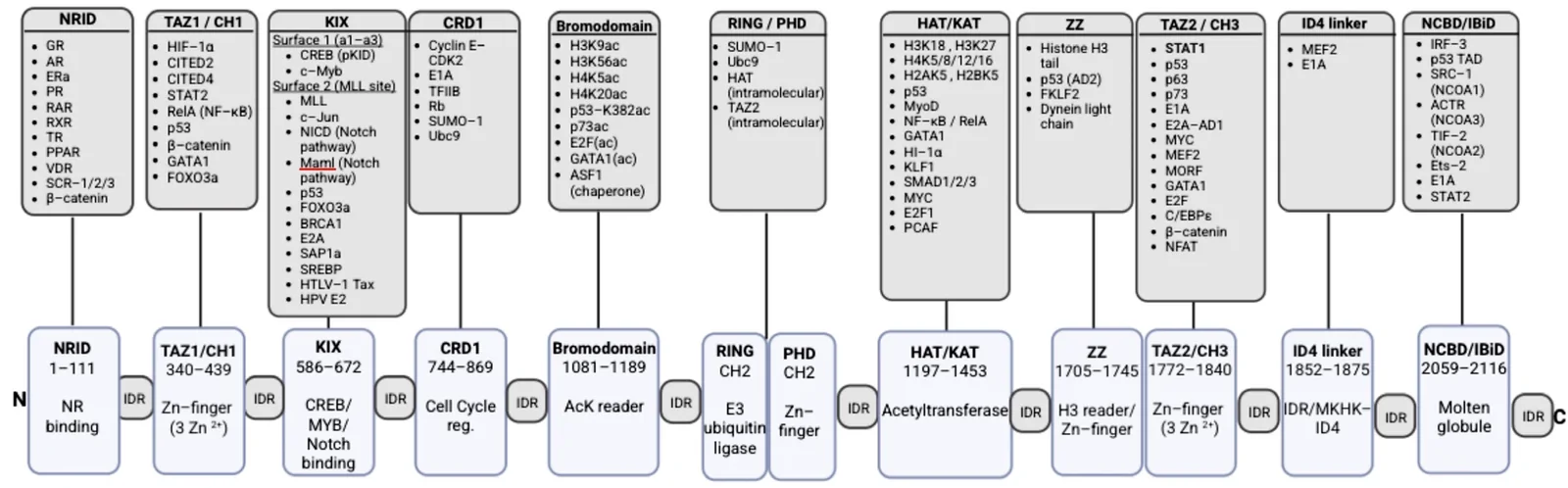
Schematic representation of the various CBP domains (NP_004371.2; not to scale) together with representative transcription factors reported to interact with the indicated domains. Domain–transcription factor interaction assignments were compiled primarily from Yazıcı and McIntyre [1] and Dyson and Wright [17], and are illustrative rather than exhaustive. CBP is shown as the representative paralog; homologous MKHK-associated ZZ, TAZ2 and ID4 regions are present in p300. Amino acid coordinates are provided in the boxes. IDR: intrinsically disordered region.

Indeed, it is now known that MKHK encompasses a broad neurodevelopmental phenotype that includes developmental delay or intellectual disability, feeding difficulties, behavioural abnormalities including autistic features, variable growth impairment and microcephaly, hearing and visual abnormalities, recurrent infections, and distinctive craniofacial features [7,8,9,10,11,13,14,15,16] (Supplementary Table S2). A major advance came from the integrated genotype–phenotype and epigenomic analysis by Haghshenas et al. [12], who studied 82 individuals with variants affecting the MKHK-associated regions of CBP or p300. The emerging clinical and epigenomic patterns aligned more closely with the affected protein domain than with gene identity alone. In particular, the disease-associated variants clustered within three structurally defined regions: the ZZ zinc-binding domain, the adjacent TAZ2 zinc-binding domain, and the first α-helical segment within the fourth intrinsically disordered linker (ID4) (Figure 1). These groups shared a common neurodevelopmental phenotype but differed in the distribution and relative prominence of individual clinical features, as well as DNA methylation patterns. Together, these findings supported re-classification of MKHK into domain-associated subtypes, MKHK-ZZ, MKHK-TAZ2 and MKHK-ID4 [12].

This domain-based organization is mechanistically important because CBP and p300 are modular regulatory proteins in which distinct regions mediate different molecular interactions and contribute differently to transcriptional and chromatin control [1,18,19,20,21]. Pathogenic variation confined to particular regulatory domains may therefore perturb selected molecular functions without producing a uniform reduction in overall coactivator activity. The clustering of MKHK variants within ZZ, TAZ2 and ID4 thus provides a structural basis for considering domain-specific disruption of CBP/p300 function as a determinant of disease heterogeneity.

Epigenomic studies add a further level of complexity. Domain-associated DNA methylation profiles have been identified in peripheral blood, while analyses of patient-derived hiPSCs indicate that the extent and pattern of methylation abnormalities vary with cellular context [12,22]. These observations link variant location and protein-domain architecture with clinical phenotype and downstream epigenomic state, while leaving unresolved how these levels are connected mechanistically.

In this narrative review, we integrate current genetic, clinical, structural and epigenomic evidence to propose a mechanistic framework for understanding MKHK. We use the term “domain-selective transcriptional rewiring” for a model in which pathogenic variants affecting discrete CBP/p300 regulatory domains perturb particular molecular interactions and thereby alter downstream transcriptional programs, rather than simply reducing coactivator function. This model generates experimentally testable hypotheses concerning variant-specific effects on protein interactions, chromatin regulation and developmental transcriptional programs, and provides a conceptual basis for selecting disease-relevant experimental systems in which these hypotheses can be evaluated.

The review was informed by a targeted search of PubMed/MEDLINE through September 2026 using combinations of the terms “Menke–Hennekam syndrome”, “CREBBP”, “EP300”, “CBP”, “p300”, “Rubinstein–Taybi syndrome”, “ZZ”, “TAZ2”, “ID4”, “DNA methylation”, “episignature”, “iPSC” and “organoid”. Primary studies reporting MKHK-associated variants, clinical phenotypes, DNA methylation findings or experimental analyses of the affected CBP/p300 regions were prioritized, together with key structural and functional studies relevant to mechanistic interpretation. Reference lists of relevant articles were screened for additional publications. This is a narrative rather than systematic review, and no formal meta-analysis or risk-of-bias assessment was performed.

## 2. Molecular Architecture and Regulatory Functions of CBP and p300

### 2.1. Enzymatic and Scaffolding Functions of KAT3 Coactivators

CBP and p300 are broadly expressed transcriptional coactivators with high sequence and structural conservation across their core functional domains. Nevertheless, they are not functionally interchangeable and both their overlapping and non-redundant functions are particularly evident during neural development and lineage differentiation [23].

CBP and p300 catalyse lysine acetylation of histones and several non-histone substrates and act as major integrators of signal-dependent transcription [1,20]. Their central regulatory region contains the bromodomain, RING/PHD region and KAT domain, while additional structured and intrinsically disordered regions mediate interactions with sequence-specific transcription factors, chromatin regulators and other signalling proteins [1,17,18,19,21,24] (Figure 1). This modular organization enables CBP and p300 to function not only as acetyltransferases, but also as chromatin readers, molecular scaffolds and interaction hubs that couple DNA-bound regulators to transcriptional machinery [1,25]. Intrinsically disordered regions further expand this interaction capacity by supporting multivalent and context-dependent molecular assemblies [17].

The traditional view of CBP/p300 primarily as histone acetyltransferases is therefore insufficient for interpreting the molecular consequences of MKHK-associated variants. A missense variant need not reduce protein abundance or abolish KAT activity to be pathogenic. Selective changes in partner binding, recruitment, interaction specificity or communication between domains may be sufficient to alter CBP/p300 regulatory function. Variants within interaction modules may also secondarily influence catalytic activity by altering substrate recruitment, chromatin engagement or communication with adjacent regulatory domains. These possibilities are particularly relevant to the ZZ–TAZ2–ID4 interval in which MKHK-associated variants cluster, but the extent to which individual variants perturb specific interactions, domain communication or catalytic output remains largely unresolved.

### 2.2. Zinc Binding and Histone Recognition by the ZZ Domain

The ZZ domain is a compact zinc-binding module immediately C-terminal to the catalytic HAT (histone acetyltransferase) region. Structural studies of CBP established an unusual fold containing two zinc ions coordinated by cysteine- and histidine-rich motifs [26]. Importantly, the ZZ and TAZ2 domains fold independently, arguing against the older view of the combined CH3 region as a single interaction unit [26]. The functional repertoire of ZZ has expanded further with work on p300 showing that the domain can recognize the unmodified N-terminus of histone H3 and facilitate selective acetylation of H3K18 and H3K27 by the adjacent HAT domain [27].

This coupling between histone recognition and catalytic activity provides a plausible reason why missense changes clustered around zinc-binding residues could have consequences disproportionate to their local size. Depending on the residue involved, a substitution might destabilize the zinc-binding fold, alter histone H3 engagement, modify interdomain communication with the HAT region, or change protein–protein interactions not yet fully mapped. At the same time, the p300 and CBP ZZ domains should not be assumed to behave identically: biochemical studies indicate differences in H3 binding between the paralogs [27]. Accordingly, sequence and structural homology support a common mechanistic framework for the two proteins, while the specific consequences of individual variants may differ between CBP and p300.

### 2.3. Transcription-Factor Recognition and Catalytic Regulation by the TAZ2 Domain

TAZ2 is a cysteine-rich zinc-binding domain composed of a compact helical bundle stabilized by three Zn^2+^ ions, each coordinated by one histidine (H) and three cysteine (C) residues in HCCC-type motifs [28,29]. The domain provides a binding platform for the transactivation domains of numerous cellular and viral proteins, including p53, STAT1, E1A and members of the C/EBP family [17,21,28,29]. These interactions are not equivalent. Many partner transactivation domains are intrinsically disordered in solution and acquire an amphipathic α-helical structure upon binding, engaging overlapping but distinct hydrophobic and electrostatic surfaces on TAZ2. Differences in helix orientation, contact residues and auxiliary interactions determine partner-specific binding modes and affinities [21,28,29]. TAZ2 therefore functions as a multivalent but structurally constrained recognition module in which relatively localized changes to the interaction surface could selectively affect recruitment of particular transcriptional regulators.

In addition to mediating partner recognition, TAZ2 contributes to regulation of the adjacent KAT domain. Biochemical and structural studies indicate that the TAZ2-containing C-terminal region can constrain p300 in a relatively autoinhibited conformation, whereas displacement or engagement of TAZ2 by transcriptional activators can facilitate catalytic activation [30,31]. These observations establish a functional connection between transcription-factor binding and acetyltransferase regulation and broaden the range of molecular consequences that may follow from pathogenic substitutions within TAZ2. Individual variants could therefore alter domain stability, partner recruitment, inter-domain communication or catalytic regulation, with the relative contribution of these effects likely to depend on the position and biochemical nature of the substitution.

The CBP p.Cys1790Arg variant we have recently described [16] provides an illustrative example of this mechanistic complexity. Cys1790 participates directly in zinc coordination within TAZ2, and its replacement by arginine is therefore expected to perturb a structurally important site, with potential consequences for domain folding and interaction surfaces. Structural modelling further suggested variant-dependent effects near a TAZ2 surface implicated in STAT1 binding [16,21] but altered CBP–STAT1 interaction and downstream STAT1-dependent transcription remain to be demonstrated experimentally. Accordingly, p.Cys1790Arg provides a tractable variant-specific hypothesis for testing in genetically matched cellular models, as discussed in Section 6.

### 2.4. Local Structure and Regulatory Potential of the ID4 Region

ID4 is often described as an intrinsically disordered linker, but this designation should not be interpreted as functional neutrality. Nuclear magnetic resonance (NMR) analysis of CBP-ID4 showed substantial alpha-helical propensity in two segments, including residues 1852–1875 of full-length CBP [32]. The first of these helices coincides with the MKHK-ID4 region identified clinically and epigenomically [12]. The local structural propensity of this segment, together with its proximity to TAZ2 and the catalytic region, raises the possibility that ID4 variants alter inter-domain communication, allosteric regulation or incompletely characterized partner interactions.

This is particularly relevant because MKHK-ID4 shows the most robust currently defined diagnostic DNA methylation episignature [12]. However, the existence of a robust episignature does not itself establish whether the initiating molecular defect is altered catalysis, altered scaffolding, aberrant recruitment, or another function. The value of the ID4 subtype is that it provides a genetically and epigenomically coherent group in which such mechanisms can now be tested.

## 3. The Genetic Architecture of Menke–Hennekam Syndrome

### 3.1. Variant Distribution, Cross-Paralog Relationships and Variant Class

**Figure 2:**
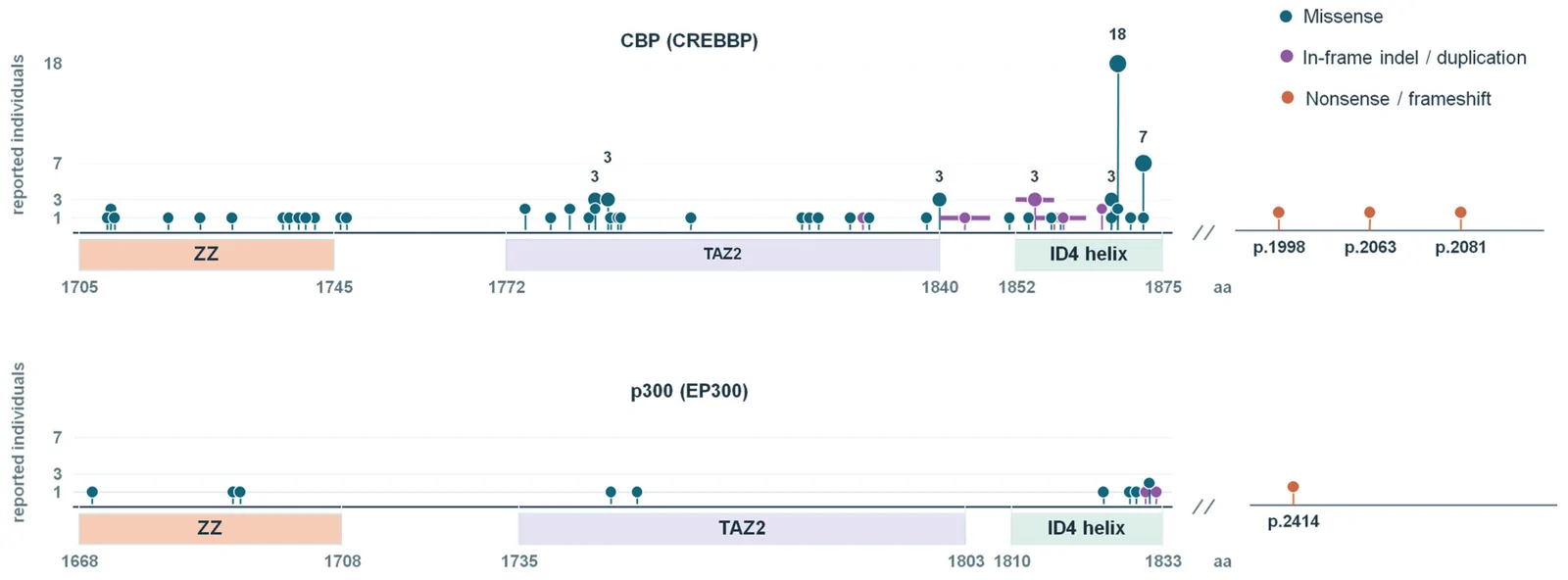
“Lollipop” diagram showing the distribution of reported *CREBBP* and *EP300* variants by protein position. Each lollipop represents a distinct cDNA variant and its height indicates the number of reported individuals carrying it (y axis). The plot includes 51 *CREBBP* variants in 90 individuals and 12 *EP300* variants in 13 individuals matched between Supplementary Tables S1 and S2. Colour indicates variant class: missense (teal), in-frame indel or duplication (purple), and nonsense or frameshift (orange). Horizontal caps indicate the residues affected by variants spanning more than one amino acid. Shaded tracks mark the ZZ and TAZ2 domains and the first α-helix of ID4. Distal variants are shown separately on an axis that is not to scale. Inclusion in the plot does not imply that every variant has established pathogenicity.

The genetic architecture of MKHK is characterized by a restricted distribution of pathogenic variants within homologous C-terminal regions of CBP and p300 (Supplementary Tables S1 and S2; Figure 2). Most established MKHK-associated variants are missense substitutions or in-frame indels affecting the ZZ and TAZ2 domains or the proximal ID4 region [7,8,9,10,11,12,13,14,15,16,22]. In the largest assembled cohort, 71 individuals carried *CREBBP* variants and 11 carried *EP300* variants, with disease-associated changes in the two paralogs clustering predominantly within corresponding functional domains [12]. This correspondence supports classification at the level of protein-domain architecture rather than gene identity alone.

Evidence that variants affecting homologous domains of CBP and p300 produce comparable clinical and epigenomic phenotypes is uneven across MKHK subtypes. Clinical and DNA methylation data provide relatively strong support for cross-paralog similarity in the ZZ and ID4 groups, whereas the TAZ2-associated methylation profile has thus far been characterized predominantly in individuals with *CREBBP* variants [12,22]. The smaller number of reported *EP300* TAZ2 variants limits assessment of whether substitutions within homologous TAZ2 positions produce comparable molecular and phenotypic consequences in the two proteins [15]. Domain homology should therefore be regarded as a basis for mechanistic comparison rather than evidence of complete functional equivalence.

Variant class introduces an additional level of complexity. The predominance of missense substitutions and in-frame indels is compatible with the production of full-length or near-full-length CBP/p300 proteins carrying localized structural or functional alterations [12]. However, the effects of individual variants on protein stability, abundance and activity have rarely been established directly. This pattern differs from the classical haploinsufficiency model of RSTS and raises the possibility that, in at least a subset of MKHK, disease results from localized alterations in CBP/p300 function rather than a simple reduction in protein dosage.

Variants in the terminal coding regions further complicate the relationship between variant class and phenotype. Nishi et al. described nonsense and frameshift variants in the terminal coding region of *CREBBP* associated with MKHK phenotypes [11]. Tang et al. subsequently reported nonsense variants p.Gln1896Ter and p.Gln1969Ter in the terminal coding region in individuals clinically classified within an atypical RSTS spectrum [22]. We assessed these two variants using NMDetective-AI [33], which predicts the efficiency of nonsense-mediated mRNA decay (NMD). Their respective scores, −0.691 and −0.690, fall below the model’s cutoff for predicted NMD escape (−0.17). The abnormal transcripts may therefore persist and produce C-terminally truncated CBP, although neither transcript abundance nor protein production has so far been measured. Whereas both cases remained positive in the RSTS DNA methylation classifier, exploratory methylation-space analyses placed their profiles outside the classical RSTS cluster and closer to MKHK-associated profiles [22]. They are therefore best regarded as atypical RSTS cases with MKHK-proximal methylation features rather than as established MKHK cases. More recently, Ketenci İşlek et al. reported a de novo *EP300* frameshift, p.His2414ThrfsTer34, that affects the protein’s terminal residue and is predicted to extend its reading frame [34], but its epigenomic and functional consequences remain unknown.

### 3.2. Recurrent Cysteine and Arginine Substitutions

The MKHK variant spectrum also shows recurrent involvement of cysteine and arginine residues. Cysteine substitutions are particularly relevant within the ZZ and TAZ2 domains because both are cysteine-rich zinc-binding modules [26,28,29]. Several cysteine residues directly coordinate Zn^2+^ and are integral to domain folding, whereas others occupy non-coordinating positions that may contribute to local structure or molecular interaction surfaces. The consequences of cysteine substitution may therefore depend on the structural role of the affected residue, the physicochemical properties of the replacement amino acid and the local molecular environment. Consequently, substitutions involving different cysteines within the same domain should not be assumed to produce equivalent effects.

The occurrence of arginine substitutions may be influenced by the increased mutability of CpG-containing arginine codons. Four of the six arginine codons belong to the CGN family and contain a CpG dinucleotide, increasing susceptibility to C>T transitions following deamination of methylated cytosine [35]. This provides a plausible contribution to some Arg-loss substitutions but has not been demonstrated to account for the MKHK variant spectrum. It also cannot explain variants such as CBP p.Cys1790Arg which results from the c.5368T>C nucleotide change [16]. Amino-acid recurrence should therefore not be assumed to reflect a shared biochemical mechanism.

## 4. Domain-Specific Genotype–Phenotype Relationships

### 4.1. Shared Clinical Features and Domain-Dependent Heterogeneity

MKHK is characterized by a shared neurodevelopmental phenotype accompanied by variable multisystem involvement (Supplementary Table S2). Developmental delay or intellectual disability is present in the great majority of affected individuals, while feeding difficulties, behavioural abnormalities including autistic features, growth impairment, microcephaly, hearing and visual abnormalities, recurrent infections and craniofacial dysmorphism occur with variable frequency [12]. The absence of the broad thumbs and halluces characteristic of RSTS remains diagnostically informative, although phenotypic overlap between the two disorders can occur.

The domain-based classification introduced by Haghshenas et al. showed that this shared clinical background is accompanied by reproducible differences in the frequency and severity of individual manifestations among MKHK-ZZ, MKHK-TAZ2 and MKHK-ID4 [12]. These differences do not define mutually exclusive syndromes, but rather domain-associated phenotypic tendencies superimposed on a common MKHK spectrum. Their interpretation requires some caution because MKHK remains rare, ascertainment is heterogeneous and several estimates are based on relatively small domain-specific groups. Nevertheless, the observed genotype–phenotype associations provide evidence that the clinical consequences of MKHK variants are influenced by the functional domain in which they occur.

### 4.2. Behavioural Prominence and Variable Neurodevelopmental Severity in MKHK-ZZ

MKHK-ZZ is generally associated with mild-to-moderate intellectual disability, although considerable interindividual variability is present [12]. Behavioural abnormalities, including autistic features, are prominent in a substantial proportion of affected individuals (Supplementary Table S2). Relative overweight was notable in the reported cohort, while hypermetropia, dental abnormalities, endocrine abnormalities, scoliosis and joint hypermobility were observed in subsets of patients. Feeding difficulties and recurrent infections also occur, although they appear less prominent than in some individuals with TAZ2- or ID4-associated disease.

The craniofacial phenotype is variable and includes thick or flared eyebrows, ptosis or blepharophimosis, a high palate and a thin upper vermilion, with sandal gaps reported in a subset of individuals [7,8,9,10,11,13,14,15,16]. The overall phenotype therefore supports recognition of MKHK-ZZ as a clinically coherent subgroup, while the breadth of manifestations indicates that domain assignment alone does not fully account for phenotypic variability.

### 4.3. Greater Neurodevelopmental Severity and Prominent Feeding Difficulties in MKHK-TAZ2

MKHK-TAZ2 tends to be associated with greater neurodevelopmental severity than MKHK-ZZ (Supplementary Table S2). Moderate-to-severe intellectual disability is frequent, and feeding difficulties are particularly prominent [12]. Short stature, microcephaly, strabismus, gastro-oesophageal reflux, hearing impairment, cryptorchidism, extremity abnormalities and recurrent infections have also been reported in substantial subsets of affected individuals. Muscle hypertrophy or hypertonia and contractures occur in some patients [12].

Compared with MKHK-ID4, the craniofacial phenotype of MKHK-TAZ2 appears less consistently recognizable, further emphasizing that domain-associated classification is not determined by facial morphology alone. Phenotypic variability is also evident among individuals carrying different substitutions within TAZ2 [14]. The CBP p.Cys1790Arg variant [16] and the recently reported *EP300* TAZ2 p.Cys1753Tyr variant [15] broaden the observed spectrum, but the limited number of such cases precludes firm conclusions regarding variant-specific or paralog-specific clinical effects. Similarly, isolated observations such as T-cell acute lymphoblastic leukaemia in an individual carrying an EP300 TAZ2 variant should not currently be interpreted as evidence for cancer predisposition in MKHK.

### 4.4. Recognizable Craniofacial Features and Variable Clinical Severity in MKHK-ID4

MKHK-ID4 is associated with one of the more recognizable craniofacial patterns within the MKHK spectrum. Reported features include a prominent forehead, sparse hair, short and upslanting palpebral fissures, ptosis or blepharophimosis, protruding upper ears, a short nose with a depressed bridge, broad nasal tip and anteverted nares, a short columella, high palate and long philtrum [12]. Intellectual disability varies in severity but is frequently moderate, and feeding difficulties remain common.

Other manifestations include constipation, hearing impairment, cardiovascular and renal abnormalities, cryptorchidism, muscle hypertrophy or hypertonia, contractures and extremity abnormalities. A recurrent *CREBBP* p.Arg1868Trp substitution has been reported within this subgroup (Figure 2) and was associated in the 2024 cohort with a higher frequency of severe intellectual disability than other ID4 variants [12]. This observation suggests that clinically relevant heterogeneity may exist not only between domains but also among variants affecting the same domain, although larger cohorts will be required to establish robust variant-level associations.

## 5. DNA Methylation and the Domain-Based Classification of MKHK

Genome-wide DNA methylation signatures have emerged as clinically useful biomarkers for a growing number of Mendelian neurodevelopmental disorders caused by disruption of chromatin regulators [36,37]. These episignatures can support molecular diagnosis, aid classification of variants of uncertain significance and reveal relationships among clinically overlapping disorders. Importantly, they are generally interpreted as downstream molecular readouts of the underlying genetic perturbation rather than direct measurements of the primary biochemical defect [36,37]. This distinction is particularly relevant to MKHK, in which DNA methylation profiles increasingly contribute to disease classification but do not themselves define the initiating molecular mechanism.

The study by Haghshenas et al. provides the strongest evidence that epigenomic variation in MKHK is associated with the affected protein domain [12]. Distinct methylation patterns were identified for variants affecting ZZ, TAZ2 and ID4, with evidence that the ZZ- and ID4-associated profiles extend across both CBP and p300. The TAZ2 profile has so far been characterized predominantly in individuals with *CREBBP* variants. The principal significance of these findings is that epigenomic classification appears to follow functional protein architecture rather than gene identity alone. This provides independent molecular support for a domain-based taxonomy of MKHK and suggests that disruption of homologous regulatory modules in CBP and p300 can be associated with related downstream epigenomic states.

The three domain-associated methylation signals differ in their consistency and diagnostic strength. The ID4 episignature showed the most consistent detection in the tested cohort, whereas the ZZ and particularly TAZ2 profiles were less consistently detected [12]. Methylation-negative and intermediate cases within TAZ2 are especially informative because they argue against a simple one-to-one relationship between domain assignment and peripheral-blood episignature. This variability may reflect differences among individual substitutions, incomplete functional equivalence between CBP and p300, developmental or cell-type effects, or technical factors including blood-cell composition and assay sensitivity. It may also indicate that variants affecting the same structural domain can perturb CBP/p300 function through different molecular routes without generating an identical stable methylation phenotype.

Tang et al. extended these observations by examining DNA methylation in patient-derived human induced pluripotent stem cells from individuals with RSTS and MKHK [22]. Disease-associated differentially methylated probes were detectable in undifferentiated hiPSCs, although fewer were identified than in peripheral blood. Direct numerical comparison between the two systems is limited by differences in cohort size and genetic composition. More importantly, reprogramming involves extensive epigenetic remodelling, and undifferentiated hiPSCs do not reproduce the developmental or cellular states most relevant to the neurological phenotype. Donor-, clone- and culture-dependent effects provide additional sources of variability. DNA methylation in hiPSCs should therefore not be regarded as a direct surrogate for the diagnostic blood episignature. Its principal experimental value lies in enabling variant-specific epigenomic changes to be followed together with transcriptional and chromatin alterations during controlled lineage differentiation.

The distal nonsense variants examined by Tang et al. further illustrate the information provided by epigenomic profiling [22]. Although the affected individuals were clinically classified within an atypical RSTS spectrum and their samples were positive in the RSTS methylation classifier, exploratory methylation-space analyses placed their profiles outside the classical RSTS cluster and closer to MKHK-associated profiles. These observations do not justify reclassification of the variants as MKHK. Whether mutant-transcript stability or the structure of any residual CBP contributes to their atypical methylation profiles remains unknown.

Taken together, the blood and hiPSC studies establish associations between variant region, cellular context and DNA methylation [12,22] but do not define a transcriptional phenotype for MKHK. An episignature identifies differences at selected CpG sites but it does not show which genes change expression in developmentally relevant cells, whether CBP/p300 occupancy or histone acetylation is altered, or whether methylation changes precede or follow transcriptional changes. Longitudinal studies in isogenic neural and neural-crest derivatives should measure these processes together. Targeted perturbation would then be needed to determine whether methylation contributes to cellular dysfunction or serves primarily as a downstream marker.

## 6. From Loss of Function to Domain-Selective Transcriptional Rewiring

### 6.1. Limits of a Uniform Loss-of-Function Model

RSTS provides a compelling example of developmental pathology caused by reduced CBP/p300 dosage or catalytic competence. Heterozygous loss-of-function *CREBBP* variants account for a large proportion of classical RSTS, and pathogenic *EP300* variants produce a substantially overlapping, often somewhat milder phenotype [2,3,4,5,6]. Mouse models with reduced CBP activity reproduce aspects of the RSTS cognitive phenotype and have supported experiments in which enhancement of cAMP–CREB signalling or histone acetylation ameliorated memory-related deficits [30,38,39]. These studies established the biological importance of CBP dosage and acetyltransferase activity but do not necessarily model MKHK.

MKHK-associated missense and in-frame variants are compatible with the production of full-length or near-full-length proteins and cluster outside the catalytic KAT active site [12]. Their restricted location within ZZ, TAZ2 and ID4 is difficult to reconcile with a model in which all pathogenic variants act mainly by quantitatively reducing total coactivator function. The clinical separation from RSTS, domain-specific methylation profiles and within-domain genotype–phenotype associations instead suggest that the functional properties retained or altered in the mutant protein contribute to disease.

This does not imply that MKHK is uniformly a gain-of-function disorder. A missense variant may reduce one interaction while enhancing another, destabilize a local fold without necessarily affecting total protein abundance, or perturb allosteric coupling between domains. The same substitution may also have different consequences across cell types if the relevant interaction partners are developmentally regulated. Accordingly, classifications such as loss of function, gain of function and dominant-negative effect are meaningful only when linked to the specific molecular activity that is altered.

### 6.2. A Domain-Selective Transcriptional Rewiring Model

We propose domain-selective transcriptional rewiring as a framework for organizing these possibilities. In this model, the affected domain defines the molecular entry point of the variant, while rewiring refers to selective alteration in regulatory interactions and downstream transcriptional outputs. The framework does not require global enhancement or suppression of CBP/p300 activity. It instead proposes that pathogenic variants alter particular molecular interactions, chromatin engagement or inter-domain regulation in ways that depend on the affected domain, individual substitution, paralog, developmental stage and cell state.

The evidence supporting this framework operates at different levels. Variant clustering within defined CBP/p300 regions and the associated clinical and DNA methylation patterns are established observations. Selective disruption of domain-mediated functions is a mechanistically plausible interpretation based on the known structural and regulatory properties of the affected regions. Domain-selective transcriptional rewiring, however, remains a hypothesis that requires direct functional demonstration in models carrying authentic MKHK variants.

The framework can therefore be tested against a simpler quantitative loss-of-function model. It would be supported by reproducible domain- or variant-selective changes in protein interactions, chromatin occupancy, histone acetylation or transcriptional output that cannot be accounted for solely by reduced CBP/p300 abundance or global KAT activity. Conversely, if variants across the ZZ, TAZ2 and ID4 regions were found to produce only graded reductions in overall CBP/p300 function, with downstream effects scaling proportionally with the magnitude of that reduction and without reproducible domain-specific molecular patterns, the explanatory value of the rewiring framework would be substantially weakened.

For MKHK-ZZ, the most direct hypotheses involve zinc-dependent fold integrity, histone H3 recognition and coupling to adjacent KAT activity [26,27]. For MKHK-TAZ2, plausible mechanisms include altered folding around zinc-binding residues, selective disruption or redirection of transcription-factor binding and changes in regulation of the adjacent KAT domain [21,28,29,31]. For MKHK-ID4, the locally structured α-helical region within an otherwise disordered linker may influence inter-domain communication or regulatory interactions [32]. These mechanisms are not mutually exclusive and may converge on partially overlapping downstream transcriptional programmes.

The concept also accommodates the observation that CBP and p300 are homologous but non-redundant. Domain-associated phenotypic and methylation patterns across both paralogs support a shared role of homologous domains in disease, particularly for ZZ and ID4 [12]. At the same time, distinct neural differentiation trajectories following loss of CBP or p300 and biochemical differences between homologous domains suggest that individual MKHK variants may exert paralog-dependent effects [23,27]. Accordingly, variants in homologous domains of CBP and p300 may have related, but not identical, molecular consequences.

The STAT1 example illustrates the appropriate level of inference. TAZ2 directly binds the STAT1 transactivation domain [21], and structural modelling of CBP p.Cys1790Arg suggested that this variant may perturb a surface relevant to that interaction [16]. In an unrelated cohesin-deficient model, increased interferon/STAT1-associated transcription accompanied neuronal abnormalities, and STAT1 knockdown partially rescued impaired synapse formation [40]. None of these observations demonstrates STAT1 dysregulation in MKHK. Together, however, they define a tractable experimental path. Genetically matched cell models differing only at the p.Cys1790Arg variant could first establish whether the substitution alters CBP–STAT1 binding. Chromatin occupancy and STAT1-dependent transcription could subsequently be examined in relevant developmental cell states. Targeted perturbation of any implicated pathway could then determine whether the molecular change contributes causally to a disease-relevant cellular phenotype. This sequence would move the hypothesis from structural plausibility to molecular validation and, ultimately, to functional causality.

## 7. Experimental Models for Resolving MKHK Mechanisms

### 7.1. Human iPSC-Derived Neural Models

**Figure 3:**
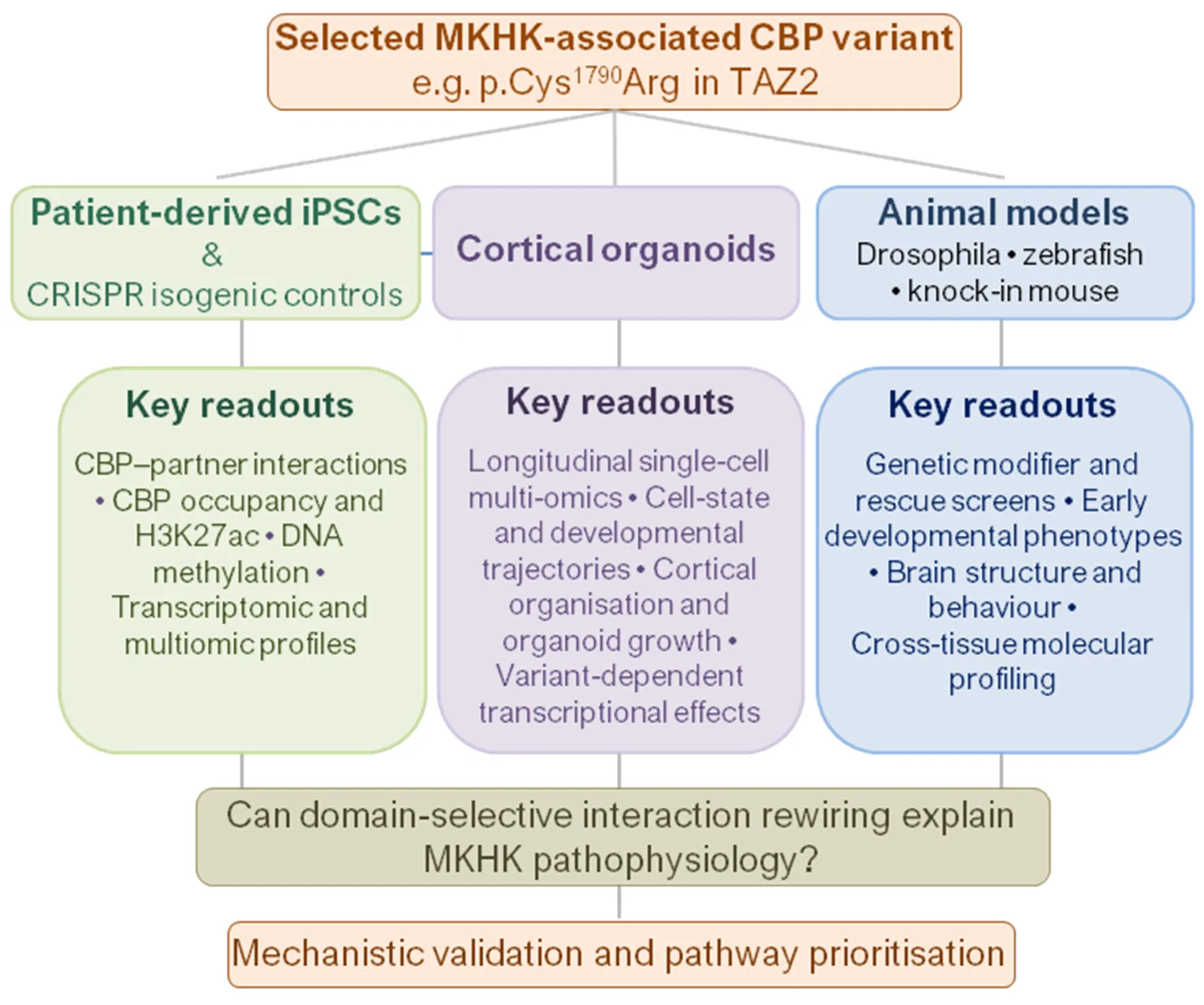
Proposed modelling framework for MKHK. Complementary platforms including patient-derived human induced pluripotent stem cell models with isogenic controls, cortical organoids, zebrafish and Drosophila models where sequence and functional conservation permit, and CRISPR knock-in mice could be used to investigate domain- and variant-dependent effects on CBP/p300-mediated regulatory networks. Representative experimental readouts are shown for each platform.

Testing domain-selective transcriptional rewiring requires experimental systems that connect a defined variant to its molecular consequences in developmentally relevant cell states (Figure 3). Patient-derived human iPSCs provide an important starting point, particularly when the pathogenic variant is corrected to generate a genetically matched control. Tang et al. showed that MKHK-derived hiPSCs retain disease-associated DNA methylation differences, although these are less extensive than those observed in blood and should not be considered equivalent to the diagnostic episignature [22]. Studies of RSTS have further established that *CREBBP*- and *EP300*-mutant iPSCs can be differentiated into neural lineages and can reveal abnormalities in neuronal morphology and excitability [41].

Undifferentiated hiPSCs are unlikely to capture the developmental consequences most relevant to MKHK. Their principal value lies in enabling controlled differentiation into neural progenitors and defined neuronal lineages, with neural crest derivatives included where the phenotype or mechanistic question justifies their use. The resulting systems can reveal effects that emerge only at particular stages of lineage commitment or maturation. Correction of a patient variant provides the most rigorous control for genetic background, while introduction of the same variant into an independent reference line can test whether the alteration is sufficient to reproduce the molecular phenotype. Independent clones, matched differentiation batches and longitudinal sampling are essential for distinguishing variant-dependent effects from experimental and background variability.

Experimental readouts should be selected according to the molecular hypothesis under investigation, integrating direct effects on protein interactions with CBP/p300 chromatin occupancy, H3K27ac and H3K18ac, chromatin accessibility and transcriptional output. Single-cell approaches will be particularly informative where variant effects are restricted to specific developmental populations or transient cell states.

### 7.2. Cortical Organoids and Developmental Timing

Cortical organoids can extend the isogenic iPSC models described above when a variant’s consequences may depend on developing tissue organization [42]. Longitudinal single-cell analyses of gene expression and chromatin accessibility could identify the earliest detectable divergence between mutant and corrected organoids and the cell populations involved. Measurements of CBP/p300 occupancy and histone acetylation in those populations could then relate the divergence to altered coactivator function. Comparing variants within the same domain would help distinguish shared from variant-specific developmental effects [12,22].

These models nevertheless have notable limitations. Organoid development varies across batches, later stages of human brain maturation are incompletely reproduced, and vascular, immune and systemic influences are largely absent. Organoids should therefore be used to address questions that depend on developmental context and tissue organization rather than as substitutes for biochemical, cellular or *in vivo* approaches.

### 7.3. In Vivo Models Across Species

Variant-specific animal models could test whether molecular changes identified in human cells alter tissue development and organismal physiology. Knock-in mice permit examination of brain organization, developmental timing and multisystem manifestations when the affected residue and molecular interface are conserved. Existing RSTS mouse models provide experimental precedents, but primarily examine reduced CBP dosage or catalytic activity [30,38,39].

Zebrafish offer complementary advantages by providing a rapid vertebrate setting for studying neural crest and craniofacial development. Perturbation of *ep300* produces developmental abnormalities relevant to KAT3-associated disease and has been used in genetic and chemical rescue studies [43]. More recent *crebbpa* and *ep300a* models, together with human iPSC-derived neural crest cells, have implicated altered neural crest migration and epithelial-to-mesenchymal transition programmes [44]. Introducing defined MKHK variants could establish whether their developmental effects converge or differ. Interpretation, however, must account for duplication of KAT3 paralogs in zebrafish and for anatomical differences from human cortical development.

*Drosophila melanogaster* offers a complementary system for dissecting conserved genetic and epigenetic mechanisms. Lineage-restricted fly models can establish causal relationships between chromatin regulation and cell identity, including Polycomb-mediated control of stem-cell maintenance and differentiation [45,46]. The fly contains a single CBP/p300 ortholog encoded by *nejire*, facilitating tissue-specific manipulation and genetic interaction studies. *Nejire/dCBP* contributes to H3K27 acetylation antagonising Polycomb-mediated repression [47], while regulation of *nejire* has also been implicated in dendrite morphogenesis [48]. Selected MKHK variants could therefore be modelled when the affected residue and relevant molecular interface are sufficiently conserved. However, fly models cannot reproduce the human-type genomic DNA methylation episignatures described in MKHK, because *Drosophila* lacks the widespread CpG methylation system that underlies mammalian episignature profiling [49].

Together, these systems could test whether the immediate molecular effect of a variant produces a developmental phenotype and determine which consequences are conserved across models.

## 8. Therapeutic Implications and Limits of Current Evidence

The distinction between RSTS and MKHK has important consequences for therapeutic reasoning. In RSTS models, reduced CBP dosage or acetyltransferase activity has provided a rationale for interventions aimed at enhancing CREB-dependent signalling or restoring histone acetylation. Phosphodiesterase-4 (PDE4) inhibition, which increases intracellular cAMP signaling by preventing cAMP degradation, improved long-term memory in a CBP-deficient mouse model [38], while HDAC inhibition ameliorated defects in histone acetylation, long-term potentiation and memory in related models [30,39]. Partial phenotypic rescue has also been reported in zebrafish following *ep300* depletion using interventions directed toward acetyltransferase-related pathways [43]. These studies establish that some consequences of reduced KAT3 activity are experimentally reversible, but they predominantly model reduced dosage or catalytic function rather than the domain-selective alterations implicated in MKHK. No pharmacological intervention has yet been validated in a variant-specific MKHK model, and therapeutic responses observed in RSTS loss-of-function models cannot be extrapolated directly to MKHK.

The therapeutic consequences of MKHK-associated variants are therefore less predictable. A variant that selectively disrupts a protein interaction, alters substrate recognition or modifies inter-domain regulation may not respond to global enhancement of CBP/p300 activity. In some circumstances, increasing acetyltransferase activity could fail to restore the affected molecular interaction and might further perturb regulatory outputs that remain intact or are already altered. Conversely, variants that destabilize a domain could impair several functions simultaneously. Similar uncertainty applies to broad HDAC inhibition, bromodomain modulation and pharmacological CBP/p300 inhibitors or degraders developed primarily in oncological settings [50]. Their effects cannot be extrapolated to MKHK without defining the molecular consequence of the relevant variant.

Therapeutic development should therefore proceed from an experimentally defined mechanism rather than from the diagnostic label alone. A candidate molecular defect should first be demonstrated reproducibly in a disease-relevant, genetically matched model and linked to a measurable developmental or cellular phenotype. Correction or targeted modulation of that defect should then produce a corresponding phenotypic improvement in a direction consistent with the proposed mechanism. Such evidence would help distinguish causal abnormalities from secondary molecular changes and would provide a rational basis for selecting therapeutic targets.

In some cases, the most tractable intervention may ultimately lie downstream of CBP/p300 rather than in the coactivator itself, particularly when a variant selectively perturbs a limited regulatory interaction or transcriptional programme. Different variants may also converge on a common downstream dependency despite having distinct primary molecular effects, while others may require domain- or variant-specific approaches. At present, however, the available evidence does not support a specific mechanism-based pharmacological strategy for MKHK. The immediate translational priority is therefore to identify reproducible molecular dependencies that can be linked to disease-relevant phenotypes and tested for reversibility.

## 9. Outstanding Questions and Experimental Priorities

The next phase of MKHK research should move beyond phenotypic delineation toward testing of molecular mechanisms.

The immediate priority is to test whether variants grouped within ZZ, TAZ2 or ID4 share molecular effects. Matched experimental models of several variants per domain, and of variants in homologous regions of CBP and p300 where available, should compare protein abundance, molecular interactions, chromatin engagement and transcriptional output. These comparisons will show whether domain assignment identifies reproducible defects or obscures variant-specific and paralog-specific effects.

A second priority is to clarify what the DNA methylation findings reveal about MKHK biology. Patient cohorts can show whether blood methylation profiles are associated with particular variants and clinical features, especially within the TAZ2 group. To investigate the underlying mechanism, representative variants could be studied in isogenic human iPSC-derived neural crest cells or neural progenitors. Measuring CBP/p300 function, gene expression and DNA methylation during differentiation could reveal when each abnormality emerges. These cells need not reproduce the blood signature, and testing whether methylation contributes to the cellular defect would require manipulating it independently of the variant.

Finally, multicentre cohorts with standardized longitudinal phenotyping and linked biospecimens are needed to determine which clinical manifestations recur within molecularly characterized groups. This will help distinguish subtype-associated features from isolated observations and assess whether molecular classification predicts clinical course.

## 10. Conclusions

Our understanding of Menke–Hennekam syndrome has evolved from the recognition of a small group of atypical *CREBBP*-associated cases to a domain-resolved neurodevelopmental disorder involving both *CREBBP* and *EP300*. The convergence of variant distribution, clinical phenotype and DNA methylation supports the existence of biologically meaningful subtypes associated with the ZZ, TAZ2 and ID4 regions. However, the evidence remains predominantly genetic, clinical and epigenomic, and the molecular events linking individual variants to altered development are still poorly defined.

This distinction should guide future mechanistic studies. Models developed for RSTS remain valuable for understanding the consequences of reduced CBP/p300 dosage and catalytic activity, but they cannot substitute for systems carrying authentic MKHK variants. Domain-selective transcriptional rewiring provides a framework for investigating whether such variants selectively alter partner interactions, substrate recognition, chromatin engagement or inter-domain regulation. These hypotheses can now be tested by combining direct biochemical analysis with genetically matched human developmental models, organoids and complementary *in vivo* systems.

More broadly, MKHK illustrates how pathogenic variation in multidomain regulatory proteins may produce selective alterations in function rather than uniform loss of activity. When disease-associated variants cluster within discrete regulatory modules, gene-level classification alone may not adequately capture their molecular consequences. Integrating protein domain, variant, paralog and developmental cell state may therefore provide a more informative basis for mechanistic classification. MKHK offers a particularly informative system in which to establish whether such domain-resolved mechanisms can connect genetic variation to epigenomic state, developmental phenotype and, ultimately, therapeutic vulnerability.

## Data Availability

No new experimental datasets were generated for this Review.

## Supplementary Materials

The following supporting information can be downloaded at: https://www.mdpi.com/article/10.3390/genes17101285/s1, Table S1: Curated published CREBBP/EP300 variant spectrum relevant to Menke–Hennekam syndrome [7,8,9,10,11,12,15,16,21,32,51,52]. Table S2: Individual-level genotypes and reported clinical features in Menke–Hennekam syndrome and related CREBBP/EP300 cases [11,12,13,14,15,16,21,32,52].
